# A Review of Dr. Hunter’s Address

**Published:** 1911-12-15

**Authors:** E. C. Kirk

**Affiliations:** Editor Dental Cosmos


### A REVIEW OF DR. HUNTER'S ADDRESS.

BY E. C. KIRK, EDITOR DENTAL COSMOS.

Comparison of the editorial review of Dr. Hunter's address published in *Current Literature* and the original text of his address at McGill University reveals a wide discrepancy in the treatment of the subject by the reviewer and the treatment of the subject by Dr. Hunter. The perversion of Dr. Hunter's meaning by the reviewer of his address, and the altogether misleading inferences which the reviewer draws from Dr. Hunter's statement, are evidently done for the purpose of pandering to the morbid appetite for sensational literature regardless of truth and accuracy, even though the public credit of a self-respecting and honorable body of professional men is incidently sacrificed. This purpose is so manifest throughout the review that it constitutes one more example of the type of sensational writing which has made the "yellow journalism" of America a byword and discredit to us both at home and abroad.

The reviewer of Dr. Hunter's address imputes to him a number of remarkable conclusions. Every one of the quotations from the *Current Literature's* editorial review of Dr. Hunter's address is wholly unwarranted by the address itself. They are inferences and conclusions read into the address by either an ignorant or a prejudiced reviewer, or both combined. We are not discussing this matter as an apologist for Dr. Hunter, but in the interests of truth and fairness, and moreover because a careful reading of Dr. Hunter's address reveals the important fact that he is keenly alive to the evils arising from the results of ill-advised and badly executed reparative dental operations done by that class of practitioners who are totally unfitted, by reason of their ignorance and lack of skill, for the practice of intelligent conservative dental work. As an example of the distinction which Dr. Hunter clearly makes between intelligent dental practice on the one hand, and ignorant and harmful dental practice on the other hand, the following quotation from his original address as published in the *Lancet* is here reproduced. He says, with respect to oral sepsis:

The problem is an important one for the dental profession, and its solution is an important one in the interests of public health, especially of our school children, thirty to fifty per cent. of whom suffer from dental and oral sepsis and its tonsillitic, pharyngeal, glandular, and other effects. For while a large body of *that profession* (italics ours) are engaged in dealing successfully with the difficult problems of dental disease and of oral sepsis, another body is no less steadily engaged in promoting sepsis of the worst character and degree by ignoring the fundamental principles connected with the anatomy, physiology, and pathology of the tissues with which they deal.

The reviewer in *Current Literature* makes this statement appear to mean that while physicians on the one hand are attempting to deal successfully with the difficult problems of dental and oral disease, the dental profession is "no less steadily engaged in promoting sepsis of the worst character," etc. The difference between the two readings is perfectly clear, and the fallacy of the reviewer's point of view and the accuracy of Dr. Huntrr's original statement are equally obvious.

The work which Dr. Hunter has done in directing the attention of his medical *confreres* to the importance of the oral cavity as a focus of infection is a work of the utmost importance, and in studying the relationships between oral sepsis and grave constitutional disorders resulting therefrom, and at the same time devoting his energies to a propaganda of the importance of oral sepsis in relation to bodily disease, so that due recognition of the gravity of the problems involved may be achieved in the minds of medical men and surgeons, he has undertaken a most laudable and worthy mission. Like all enthusiasts who have hit upon a truth in the realm of scientific investigation, Dr. Hunter is evidently so absorbed with the possibilities of his special line of investigation that he is to a notable degree prejudiced in favor of his own viewpoint.

He announces himself as the discoverer of oral sepsis. We have no disposition to deprive him of any personal satisfaction which he may derive from that belief, but inasmuch as a number of observers had already reported upon the general principles of mouth infection before Dr. Hunter had announced his oral sepsis theory in July 1900, it is a contravention of history for Dr. Hunter to lay claim to priority of discovery of the septic influence of mouth infection, in principle at least, although it may be possible that the phrase "oral sepsis" may have been coined by him to describe the condition, as he claims. The whole of Miller's work on the "Micro-organisms of the Human Mouth" is a preliminary study of this question, and Miller's subsequent articles appearing in the *Dental Cosmos* under the title "The Human Mouth as a Focus of Infection," during the year 1891, are a continuation of the same line of research. The dental profession has therefore been in possession of the fundamental principles of oral sepsis for a considerable period of time, and before any of Dr. Hunter's observations and utterances were brought to the notice of either the medical or the dental profession. Moreover the dental profession—or, more strictly speaking, that portion of it that may be properly designated as the dental profession—had already, in the light of the pioneer researches of Miller, Black and others, modified its practice in accordance with the principles of "oral sepsis" years before the importance of the subject was rediscovered by Dr. Hunter.

On the other hand, it is equally true—and Dr. Hunter in his address is evidently in accord therewith—that septic infection arising in the mouth is a condition which has been practically ignored both by physicians and surgeons almost entirely up to the present time. It is only recently that it has been possible for dental pathologists, and those practitioners who were conversant with the important bearing which oral sepsis has upon general bodily health, to secure from medical men or surgeons a sympathetic hearing of their demands for a recognition of the importance of oral sepsis in medical and surgical work. It is only within very recent times, and through the insistence of well-informed dental practitioners, that surgeons were willing to concede the necessity for a thorough mouth sterilization as a part of the routine technique of preparation of patients for surgical operation, even in cases where the procedure involved operation upon the gastro-intestinal tract. It has been until recently a common procedure to enforce most rigid antiseptic technique involving the sterilization of everything pertaining to the operation—with the exception of the infected mouth of the patient, which in certain instances was continually pouring unnumbered pathogenic bacteria into the immediate fields of operation throughout the whole period of repair. Through the insistence of well-informed dental pathologists this surgical inconsistency has been to a large degree remedied in the United States, the home of that American dentistry which forms such an important factor in Dr. Hunter's discussion.

Dr. Hunter frequently refers to his extensive experience, and we do not doubt that it has been a large one, but unfortunately for the logical importance of some of his criticisms of "so-called American dentistry," the illustrative cases which he cites in his address are with a single exception cases in which no dentistry at all had been done, but on the contrary were typical cases of absolute dental neglect. The only case whose history he cites at length in which any dentistry had been done is a case of septic gastritis in which the patient had been wearing "a tooth-plate in his upper jaw which he had not removed for two and a half years, which he was told not to remove, and which even now he could only remove with difficulty and after persuasion." As the record of the case states that the man "was admitted," we assume that he was admitted either to the Charing Cross Hospital or the London Fever Hospital, in both of which institutions Dr. Hunter is attending physician. Therefore in the only case which Dr. Hunter cites among his "horrible examples" of oral sepsis in connection with constructive dental appliances it was probably not a case of American dentistry at all, nor probably was it an American dentist who told the patient "not to remove his artificial plate," but the case of a charity hospital patient who, as is typical of his class, had doubtless consulted a cheap advertising quack, a "so-called American dentist," who had never seen America and of whose cult London is full.

Now a final word with reference to Dr. Hunter's use of the term "American dentist." To his credit be it said that each use of this phrase by him appears in quotation marks. It is historically true that modern crown and bridge work is mainly a development of American ingenuity, and that in its better expressions the work has been of such value in the restoration of the masticating mechanism and a consequent improvement to health that it has come to be recognized as a valuable restorative measure, and because of the attention which has been paid to it in this country, the appellation of "American dentistry" or "American method" has been used to designate the particular class of work which has found universal acceptance. Unfortunately, however, Dr. Hunter in his enthusiasm for his cause has failed to make as plain as he should make it the distinction which he has clearly implied between such work skillfully executed and intelligently applied and the monstrous anatomical and physiological insults which are palmed off upon an ignorant public by equally ignorant charlatans under the general term of American crown and bridge work. Everyone knows or ought to know that throughout the length and breadth of the civilized world there are incompetant charlatans who are exploiting their ignorance and lack of mechanical skill in the production of ill-fitting dental mechanisms productive of eactly the conditions that Dr. Hunter describes, and that in order to catch the credulous these professional mountebanks advertise themselves as "American dentists." Every dental practitioner knows that untold suffering is inflicted upon humanity, disease is propagated, and death in many instances hastened, by the evil effects of this class of work. We infer from a careful reading of Dr. Hunter's address that it is this particular class of practitioner and the damage which he inflicts upon humanity that has enlisted his interest and called forth his critical condemnation —in which cause we wish him God-speed and more power to his arm.

Dr. Hunter's putting of the case would, however, have been more judicial, and withal more equitable, had he made it clear that he had some practical knowledge from personal observation of what really good dental work means as a conservator of health. In so far as he expresses himself upon the effects of oral sepsis upon the general bodily health he speaks with authority, and none too strongly. On the other hand, when he endeavors to discuss both the pathology and the treatment of the local lesions involved in oral sepsis, he is evidently "talking without his book" and dealing with a subject with which he is decidedly less familiar. That oral sepsis can be combated by swabbing the infected tissues occasionally with a one- in-sixty aqueous solution of carbolic acid, and the occasional "tilting off" of a "scab or crust" of tarter is a mode of dealing with the situation which all well-informed dental practitioners will regard as extremely primitive, to say the least.

Dr. Hunter's address, and more particularly the distorted review of his address by the writer in August *Current Literature*, has aroused much comment and not a little tart criticism from members of the dental profession. While some of the statements made by Dr. Hunter are perhaps rather excessive, they are on the whole, we believe, in accordance with the general facts of the situation. Though the truth is frequently unpleasant, it is never unwholesome, and if the stimulus of Dr. Hunter's pointed criticism shall arouse the organized dental profession to renewed activity in the effort to eradicate the ignorant and incompetent practice of dentistry, he will have conferred a boon upon humanity.

We are glad that Dr. Hunter has been able to arouse an interest in the question of oral sepsis upon the part of his medical colleagues. His next effort, in order to furnish himself for the further prosecution of his task, should be to make a careful study of the possibilities of "conservative dentistry," about which he seems to know very little. He has expressed his opinion very definitely with respect to the defects of "American dentistry," and the effects of this defective American dentistry upon the health of the American public as compared with the health of the people of his own nation. It is evident that he has visited America, but that he has ever investigated the conditions of dental education in America, or that he knows anything at all about what is really conservative American dentistry, does not appear either in his address or in any record that he has left of his American visit. That the teeth of the American public are any more defective than those of other nations is questionable. Indeed with respect to the teeth of the British public, or at least that portion of it which would come to the notice of Dr. Hunter in his hospital work, it has been said by Sir William Osler, an English medical authority of no less importance than is Dr. Hunter, that the negledct of the teeth of such people in England "is a national disgrace," and it may therefore be possible that Dr. Hunter has read into his American observations something of his home impressions. —*Dental Cosmos* for October.

